# Comparative Health Assessment of *Crassostrea belcheri* from Breeding and Farming Sites in Thailand: Histopathological, Apoptotic, and Molecular Evidence

**DOI:** 10.3390/ijms27125351

**Published:** 2026-06-13

**Authors:** Supatcha Chooseangjaew, Suwat Tanyaros, Narit Thaochan, Sirilak Dusitsittipon, Natthawut Charoenphon, Gen Kaneko, Supapong Imsonpang, Nabhasbhichayabha Daewang, Kitipong Angsujinda, Kitiya Kongthong, Sinlapachai Senarat

**Affiliations:** 1 Aquaculture Technology Program, Faculty of Science and Fisheries Technology, Rajamangala University of Technology Srivijaya, Trang 92150, Thailand; supatcha.c@rmutsv.ac.th; 2 Marine Science Program, Faculty of Science and Fisheries Technology, Rajamangala University of Technology Srivijaya, Trang 92150, Thailand; suwat.t@rmutsv.ac.th; 3 Agricultural Innovation and Management Division, Faculty of Natural Resource, Prince of Songkla University, Songkhla 90110, Thailand; narit.t@psu.ac.th; 4 Department of Parasitology and Entomology, Faculty of Public Health, Mahidol University, Bangkok 10400, Thailand; sirilak.dus@mahidol.ac.th; 5 Department of Anatomy, Faculty of Medical Science, Naresuan University, Phitsanulok 65000, Thailand; natthawutch@nu.ac.th; 6 College of Natural and Applied Science, University of Houston-Victoria, Victoria, TX 77901, USA; gkaneko2@gmail.com; 7 Division of Health and Applied Sciences, Faculty of Science, Prince of Songkla University, Songkhla 90110, Thailand; supapong.i@psu.ac.th; 8 Department of Microbiology, Faculty of Science, Chulalongkorn University, Bangkok 10330, Thailand; 6678028923@student.chula.ac.th; 9 Aquatic Resources Research Institute, Chulalongkorn University, Bangkok 10330, Thailand; kitipong.an@chula.ac.th; 10 Faculty of Medical Science, Naresuan University, Phitsanulok 65000, Thailand; kitiyak67@nu.ac.th; 11 Division of Biological Science, Faculty of Science, Prince of Songkla University, Songkhla 90110, Thailand; 12 Center for Integrative Animal Tissue Biology and Innovation (iTissue-PSU), Prince of Songkla University, Songkhla 90110, Thailand

**Keywords:** aquaculture, environmental stress, *Crassostrea belcheri*, white scar oyster, histopathology, apoptosis, TUNEL assay

## Abstract

Oyster health is important for aquaculture productivity and sustainability. In Thailand, the white scar oyster, *Crassostrea belcheri*, is being promoted for cultivation, yet its health status has not been compared between research breeding and community farming sites. This study evaluated histopathological features, ultrastructure, apoptosis, and defender against apoptotic death 1 (*dad1*) gene expression in sexually mature *C. belcheri* collected from these two sites. Gill tissues were examined by histology, transmission electron microscopy, TUNEL assay, and gene expression analysis, while organ condition was assessed using a Health Assessment Index (HAI). The proportion of TUNEL-positive cells in the gills and mantle differed significantly between sites (*p* < 0.05), with higher levels in oysters from the farming site. In contrast, TUNEL-positive cells in the digestive gland did not differ significantly between sites, although brown cells were observed only in the digestive gland of oysters from the breeding site, suggesting possible physiological stress. To assess the expression level of *dad1* in oysters cultured under different conditions, RT-qPCR revealed no significant difference between the two sites. The breeding site also had lower temperature and salinity than the farming site. Overall, these findings suggest that site-specific environmental conditions may influence gill health and stress-related responses in *C. belcheri*, providing baseline information for oyster health assessment and aquaculture management.

## 1. Introduction

Aquaculture has increasingly been recognized as a significant source of high-quality protein for human consumption. Optimal aquaculture conditions and hatchery technologies are continuously evolving [1,2]. In the case of oysters, seeds are produced year-round with consistent quality and sufficient quantity to meet current market demand [1,2,3]. However, oysters must cope with environmental stressors, such as fluctuations in water temperature and salinity, stock density, regular handling, and nutritional deficiency. These factors are challenging to manage and can affect their immune system, germ cell development, and reproductive efficiency [1,4,5]. Further technological development would improve oyster broodstock health, fertility, and the survival rate of the seed [1].

The gill is a vital organ in bivalves that directly contacts water and responds to environmental changes [6,7]. Gill histology thus provides valuable insights into monitoring bivalve health in both field and laboratory conditions [8,9]. The intricate network of gill lamellae is highly susceptible to stressful conditions and toxicants [8,10], and histopathological alterations of gill morphology are known as biomarkers of stress. In the Al-Khiran Coast of Kuwait, for example, *Pinctada radiata* exposed to polycyclic aromatic hydrocarbons exhibited necrosis and edemas of branchial lamellae, indicating a significant impact on their health [10]. Histopathological changes in these vital tissues have been widely used in programs that monitor oyster health under different environments [11,12].

Oxidative stress occurs when an organism’s antioxidant systems are unable to neutralize reactive oxygen species (ROS) effectively, leading to cellular damage [13,14]. There are several common markers widely used to indicate oxidative stress. Malondialdehyde (MDA) indicates the peroxidation of lipids, while superoxide dismutase (SOD) is a crucial enzyme involved in detoxifying ROS [15,16,17]. Under high ROS-induced oxidative stress, cells may trigger apoptotic pathways, which can be identified by the Terminal deoxynucleotidyl transferase dUTP Nick-End Labeling (TUNEL) assay [18]. Apoptotic cells serve as a marker in various organisms, including oysters and other bivalves under environmental stress [18,19,20]. Therefore, evaluating TUNEL-positive cells is a highly regulated process of cellular responses to environmental stress and can be used to signify the indicator of organismal health status [20,21]. Although caspase genes are widely used as apoptotic markers, the present study focused on defender against apoptotic death 1 (*dad1*), which plays a critical role in suppressing apoptosis signaling pathways [21]. This gene can therefore serve as an appropriate molecular marker for assessing apoptosis-related cellular responses [22]. Furthermore, previous studies found that *dad1* expression is associated with cellular integrity, physiological condition, and immune responses in marine invertebrates [21,22]. As mentioned above, *dad1* is a suitable molecular marker for precise cell death detection.

The white-scar oyster, *Crassostrea belcheri*, is an economically important bivalve species for aquaculture in Thailand due to its fast growth rate and high production, which significantly contribute to the development of the coastal economy [3]. Currently, the major cultivation site for *C. belcheri* is the Marine Shellfish Breeding Research Unit, Faculty of Science and Fisheries Technology, Rajamangala University of Technology Srivijaya (RUTS), Trang, Thailand [23,24,25]. Their oyster seed production has generally been successful due to artificial breeding techniques in hatcheries [24], the application of gamete stripping methods [25], and the formulation of specialized diets using mixtures of single-celled algae [26]. One of the major challenges in developing aquaculture systems for *C. belcheri* is maintaining the oyster’s health and condition during their transfer from the breeding to farming sites. Nowadays, seedlings and sexually mature *C. belcheri* from the breeding site are transferred to farming sites along the Andaman Sea, Thailand, through community-based management programs. However, there remains a lack of comprehensive data on the condition of *C. belcheri* at the time of this transfer. Only Sagulsawasdipan et al. [3] reported a few histopathological changes in captive *C. belcheri*. For these reasons, the present study assessed the health of *C. belcheri* at breeding and farming sites using histological analysis, transmission electron microscopy (TEM) examination, the TUNEL assay, and *dad1* gene expression in the gill. Moreover, oxidative stress levels were assessed using MDA and SOD as markers in the whole tissues of *C. belcheri* from the two sites. Integrated methods from these objectives were followed by the standard protocols [27,28,29,30,31].

## 2. Results

### 2.1. Water Quality Parameters Between Breeding and Farming Sites

Water temperature and salinity were significantly higher at the farming site than at the breeding site, whereas pH and dissolved oxygen (DO) were significantly lower at the farming site. In contrast, nitrite, ammonia, and total alkalinity did not differ significantly between sites (Table 1).

### 2.2. Gill Structure and Its Ultrastructure, Together with the Appearance of TUNEL-Positive Cells

**Figure 1:**
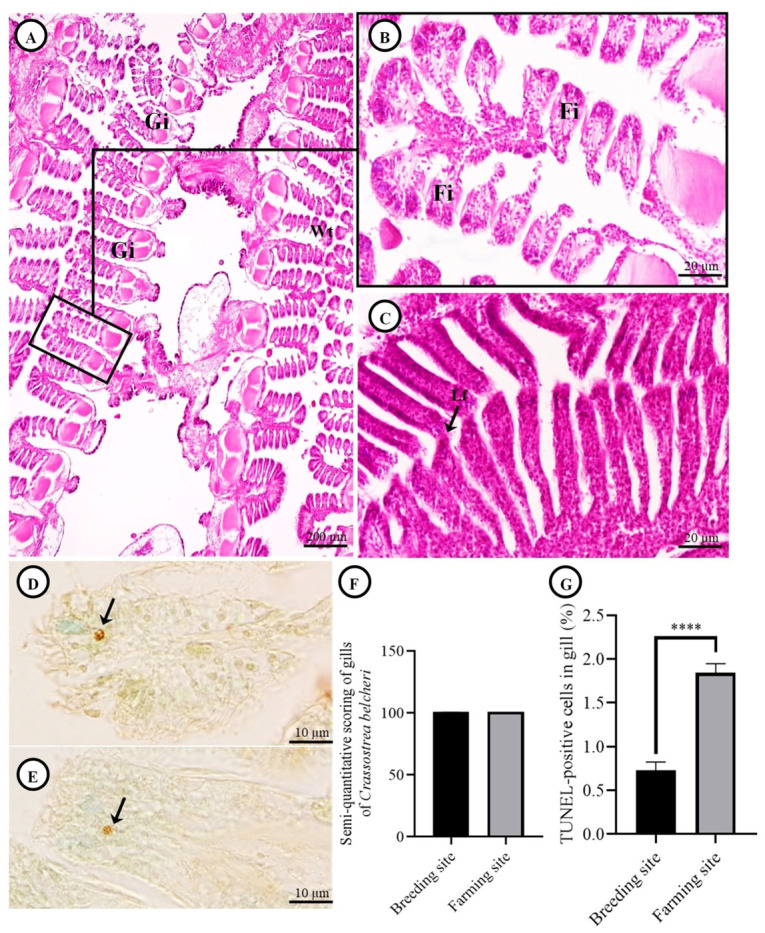
Histological analysis of gill tissues in *Crassostrea belcheri* from farming and breeding sites. (**A**) A low-magnification view of the gill (Gi) shows the central axis of filaments (Fi): (**B**) A higher magnification of several lamellae of filaments (Fi). (**C**) Highlighted histopathological changes include lamellar fusion (Lf). (**D**,**E**) A TUNEL-positive cell is indicated by the arrow. (**F**) Semi-quantitative scoring of histopathological alteration indexes for gill tissues of oysters from the breeding and farming sites. (**G**) The proportion of TUNEL-positive cells in the gills of oysters from the breeding and farming sites. Statistically significant differences between breeding and farming sites are denoted by asterisks (**** *p* < 0.0001). Abbreviations: Wt = water tube.

The gill architecture of *C. belcheri* was structurally similar across the two sampling sites and consisted of a central axis surrounded by water tubes (Figure 1A). Each gill had several gill filaments (lamellae), which were covered by the gill epithelium, terminating at the apical cilia (Figure 1B). Lamellar degeneration and fusion (Figure 1C) were the only histopathological alterations identified in oysters from both sites. TUNEL-positive cells were found in both breeding (Figure 1D) and farming (Figure 1E) sites. The HAI values indicated severe histological damage in oysters from the breeding site, with a mean score of 100.9 ± 0.36 (Figure 1F), compared with 101.0 ± 0.00 at the farming site (F = 19, *p* > 0.05). However, the TUNEL assay in the farming site revealed a significantly higher proportion of TUNEL-positive cells in gill tissues (Figure 1G) from the farming site (1.84 ± 0.1%) compared with the breeding site (0.73 ± 0.09%). Statistical analysis confirmed a significant difference between sites (F = 1.202, **** *p* < 0.0001, Figure 1G).

**Figure 2:**
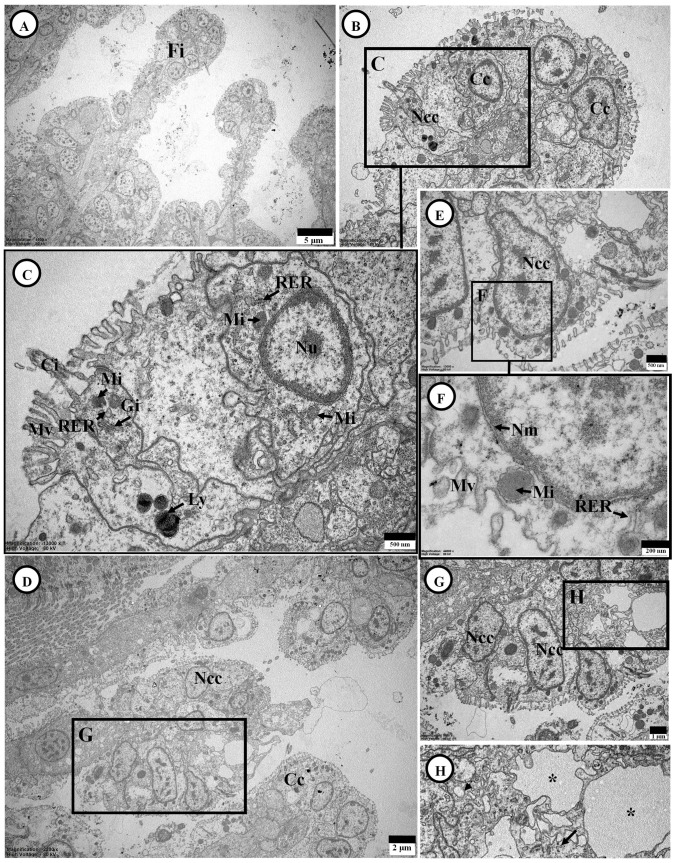
The gill ultrastructure of representative *Crassostrea belcheri* from the breeding site. (**A**,**B**) Gill filaments (Fi), non-ciliated epithelial cells (Ncc), and ciliated epithelial cells (Cc). (**C**,**D**) A high-magnification image showing Ncc without cilia (Ci) but with microvilli (Mv) at the apical surface, and presenting rough endoplasmic reticulum (RER), lysosome (Ly), Golgi apparatus (Gi), and many round or ovoid mitochondria (Mi). (**E**,**F**) High-magnification images showing Cc with cilia and organelles observed in the Ncc. (**G**,**H**) Some non-ciliated epithelial cells with large and irregular shapes (asterisks) surrounded by secretory granules of varying sizes (arrows). Abbreviations Nm = nuclear membrane, Nu = nucleus.

**Figure 3:**
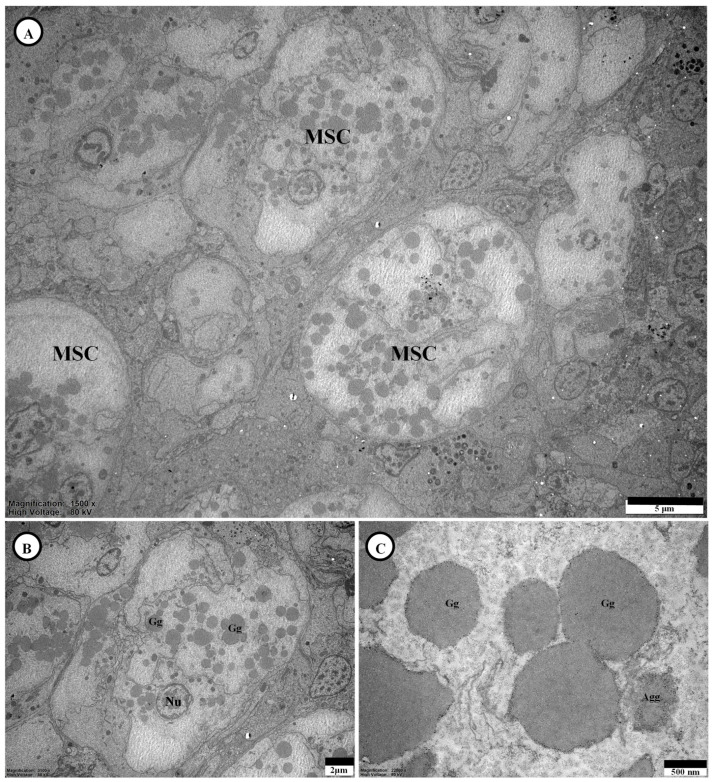
The ultrastructure of mucous-secreting cells (MSC) of *Crassostrea belcheri* with many granular granules (Gg) from breeding site (**A**,**B**): an abnormal granular granule (Ag) is also present (**C**). Abbreviation: Nu = nucleus.

According to the TEM images, gill ultrastructure was similar between the two sites. The fine structural characteristics of gill filaments (Figure 2A) were lined by prominent epithelium, and cells were classified into three distinct cell types, including non-ciliated epithelial cells (Ncc), ciliated epithelial cells (Cc), and mucous-secreting cells (MSC) (Figure 2B–H and Figure 3). Several organelles, including rough endoplasmic reticulum (RER), Golgi apparatus (Gi), lysosome (Ly), and many round or ovoid mitochondria, especially located in the apical portion of the cell, were observed in the Ncc (Figure 2C). The Cc was easily identified from the presence of cilia and organelles, as in the case of the Ncc (Figure 2C–F). Some Nccs were filled with large and irregularly shaped round secretory granules of varying sizes and of low electron densities, which were only found in specimens from the breeding site (Figure 2D–G). Some NCCs with large and irregular shapes were surrounded by secretory granules of varying sizes (Figure 2H). The MSCs contained many electron-dense granules and showed few signs of an abnormal granular granule (Figure 3A–C).

### 2.3. Molecular Identification and Expression Profile of dad1 in Gill Tissues

**Figure 4:**
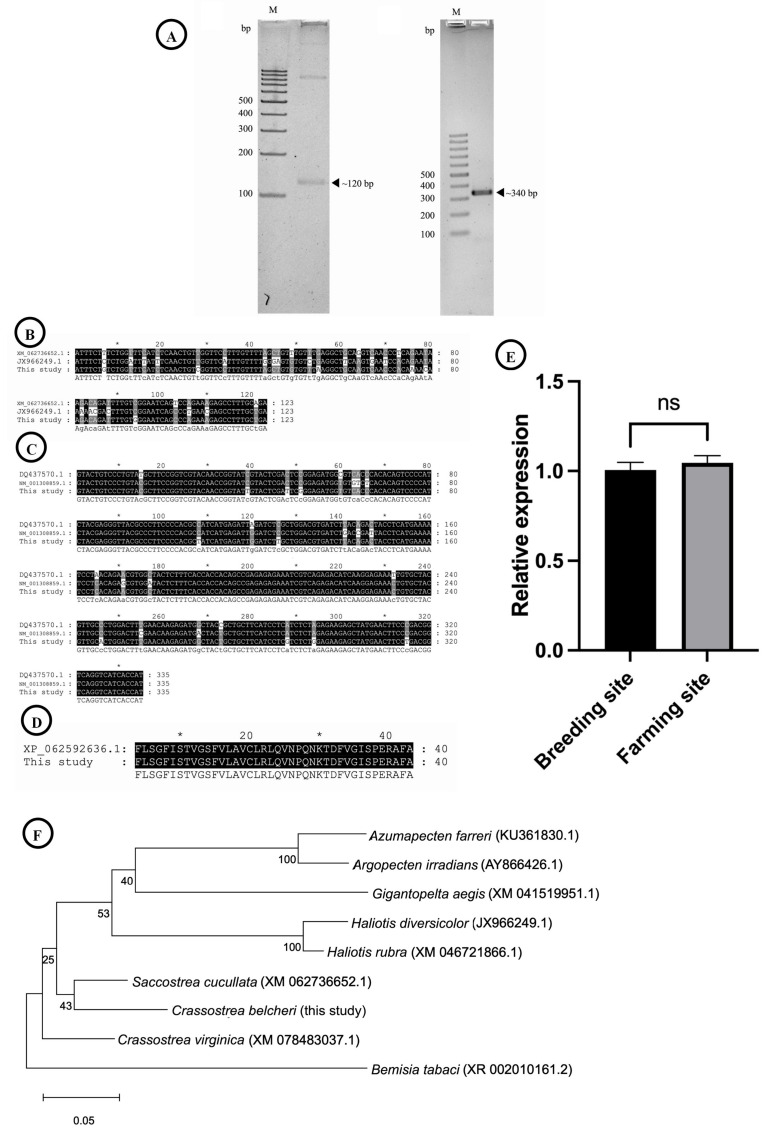
Molecular characterization, expression analysis, and phylogenetic placement of the partial *dad1* fragment in *Crassostrea belcheri*. Colony PCR was used to amplify the *dad1* and *β-actin* gene fragments of *C. belcheri* from recombinant *E. coli* DH5α colonies containing plasmids harboring either the *dad1* or *β-actin* gene fragment. (**A**) Polyacrylamide gel electrophoresis showing the *dad1* amplicon at approximately 120 bp and agarose gel electrophoresis showing the *β-actin* amplicon at approximately 340 bp. Lane M: GeneRuler 100 bp DNA ladder (Thermo Fisher Scientific, Waltham, MA, USA). (**B**) Nucleotide sequence alignment of the *C. belcheri dad1* fragment with homologous sequences from other mollusks, including *dad1*-like from *Saccostrea cucullata* (XM_062736652.1) and *dad1* from *Haliotis diversicolor* (JX966249.1). (**C**) Nucleotide sequence alignment of the *C. belcheri β-actin* fragment with homologous sequences from related species, including *β-actin* from *C. ariakensis* (DQ437570.1) and *actin* from *Magallana gigas* (NM_001308859.1). (**D**) Amino acid sequence alignment of the translated partial *dad1* fragment from *C. belcheri* with homologous DAD1 proteins. The translated sequence showed 100% amino acid identity with the DAD1-like protein of *Saccostrea cucullata* (XP_062592636.1) across the aligned region, supporting the correct reading frame of the amplified fragment. Asterisks above the alignments in panels (**B**–**D**) indicate 10-position interval markers (nucleotides in panels (**B**,**C**) and amino acid residues in panel (**D**)). (**E**) Relative *dad1* expression levels in gill tissues of *C*. *belcheri* specimens from the two sampling sites, as determined by RT-qPCR using *β-actin* as the reference gene. (**F**) Phylogenetic analysis of partial *dad1* nucleotide sequences from *C. belcheri* and representative molluscan species using the Neighbor-Joining method. Evolutionary distances were calculated using the Maximum Composite Likelihood model, and branch support was assessed with 1000 bootstrap replicates. The analysis was performed using MEGA X, with *Bemisia tabaci* as the outgroup. The cloned *C. belcheri dad1* fragment clustered within the molluscan *dad1* lineage and grouped with oyster *dad1* homologs, including *Saccostrea cucullata* and *Crassostrea virginica*, with approximately 79–91% nucleotide sequence identity across the aligned region.

RNA extracted from the gill tissue of *C. belcheri* was used for PCR. The resulting amplicons were cloned into plasmid vectors for sequence analysis. Colony PCR revealed amplicons of approximately 120 bp for *dad1* and 340 bp for *β-actin* (Figure 4A), corresponding to the expected product sizes.

Subsequent sequence alignment confirmed the identity of the *dad1* fragment, which shared 91.1% nucleotide identity with the *dad1*-like gene of *Saccostrea cucullata* (XM_062736652.1) and 81.3% with *dad1* of *Haliotis diversicolor* (JX966249.1) (Figure 4B). Moreover, translation of the correctly framed nucleotide sequence revealed 100% amino acid identity with the DAD1-like protein of *S. cucullata* (XP_062592636.1) across the aligned region, supporting the correct reading frame of the amplified fragment (Figure 4D). The *β-actin* fragment showed 95.5% identity to *β-actin* of *C. ariakensis* (DQ437570.1) and 94.3% to *actin* of *Magallana gigas* (NM_001308859.1) (Figure 4C). These findings confirmed the successful amplification of the target genes.

To assess the profile of *dad1* in oysters cultured under different conditions, RT-qPCR was performed using *β-actin* as a reference gene. The analysis revealed no significant difference in *dad1* expression levels in gill tissues between the two sites (Figure 4E).

Phylogenetic analysis demonstrated that the cloned partial *dad1* fragment from *C. belcheri* clustered within the molluscan *dad1* lineage and was separated from the insect outgroup, *Bemisia tabaci*. The *C. belcheri dad1* fragment grouped with oyster *dad1* homologs from *S. cucullata* and *C. virginica* and shared approximately 79–91% nucleotide sequence identity with other molluscan *dad1* sequences across the aligned region. These results support the identification of the cloned fragment as a partial *dad1* homolog from *C. belcheri* (Figure 4F).

### 2.4. Histology and Histopathology Assessment of Selected Organs Associated with the Appearance of TUNEL-Positive Cells

**Figure 5:**
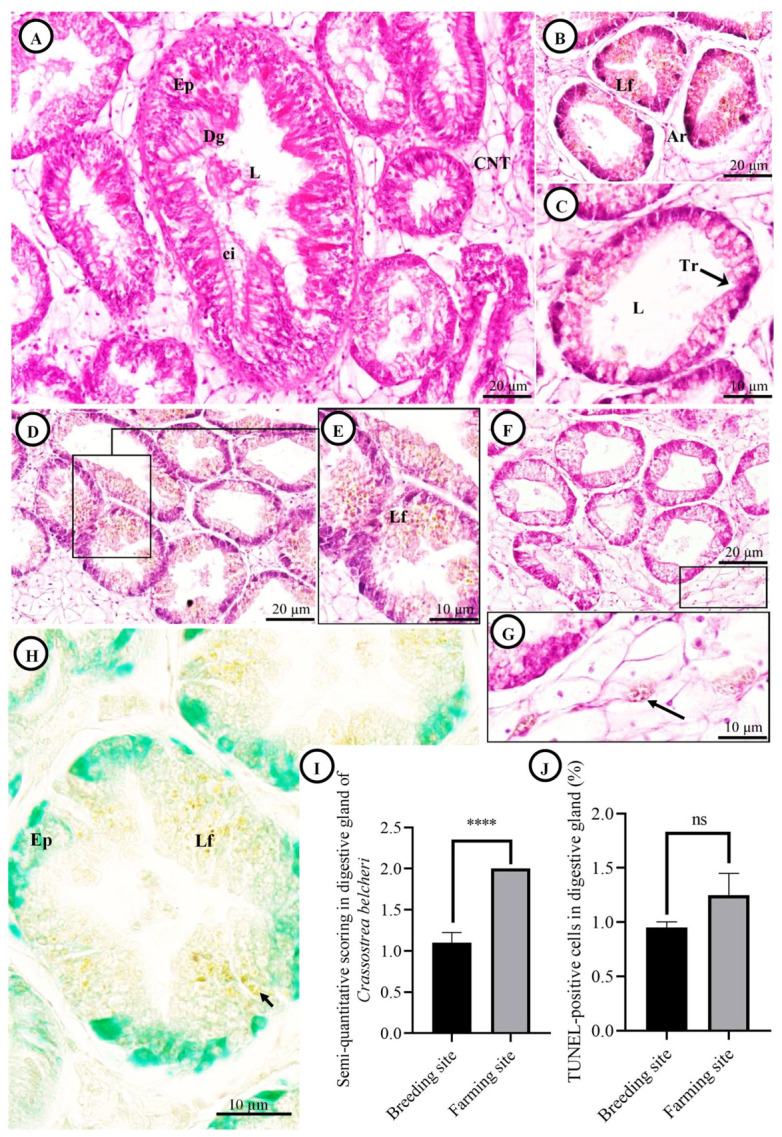
Histopathological assessment of the digestive gland (Dg) of *Crassostrea belcheri* from breeding and farming sites. (**A**) Anatomical features of the Dg include epithelium (Ep), digestive gland (Dg), lumen (L), connective tissue (CNT), and cilia (ci). (**B**–**E**) High-magnification views of the tissue showing histological alterations of atrophy (Ar), tubular regression (Tr) and lipofuscin (Lf). (**F**,**G**) The presence of brown cells (arrow). (**H**) TUNEL-positive cells indicated with arrows. (**I**) Semi-quantitative scoring of histological features in the digestive gland of *C. belcheri*, comparing specimens from the breeding and farming sites. Significant differences are denoted by asterisks (**** *p* < 0.0001) (**J**) No significant difference was observed in the percentage of TUNEL-positive cells. Abbreviations: L = lumen.

The digestive gland of *C. belcheri* from both sites exhibited a well-organized structure and was composed of epithelial-lined glands surrounding the central lumen (Figure 5A). Cross-sectioned digestive glands were infiltrated with MSC (Figure 5B). The digestive tube was located close to the digestive gland (Figure 5A). Histopathological alterations predominantly observed in digestive cells from both sites included brown cells and lipofuscin accumulation in the cytoplasm (Figure 5C–H). The HAI was significantly different between the two sites (1.1 ± 0.55 for breeding site and 2.0 ± 0.0 for farming site, F = 19, **** *p* < 0.0001, Figure 5I). On the other hand, the TUNEL-positive cells were evident within the digestive epithelium (Figure 5H). Quantitative analysis showed a higher proportion of TUNEL-positive cells in the digestive tubules of oysters from the farming site than in those from the breeding site, which was not statistically significant (Figure 5J).

**Figure 6:**
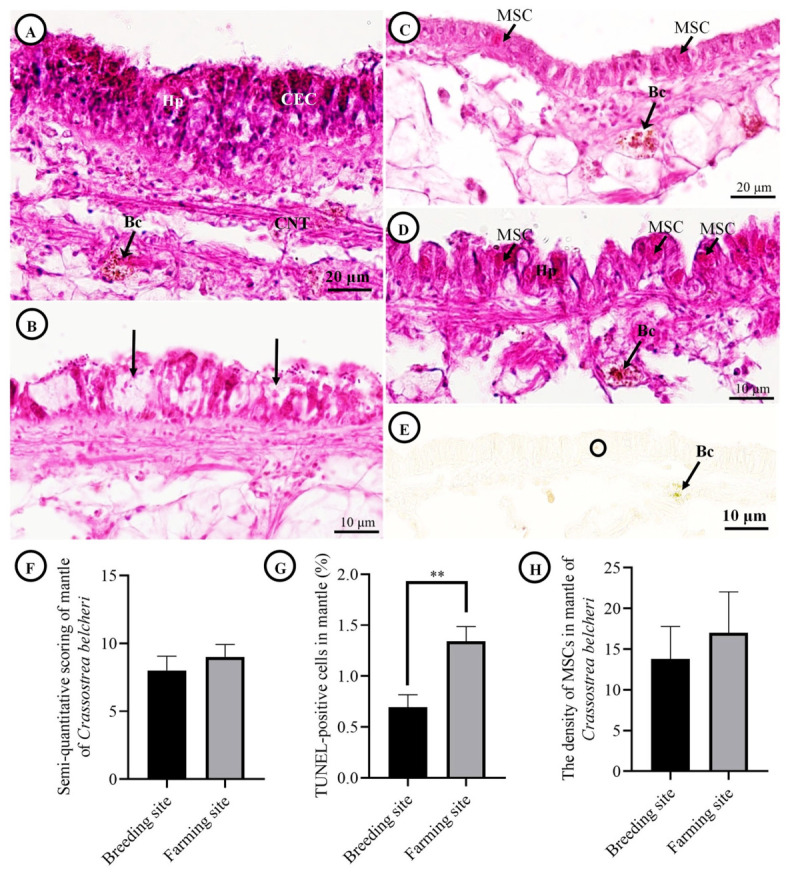
Histological analysis of mantle in *Crassostrea belcheri* from breeding and farming sites. (**A**,**B**) The mantle is lined with columnar epithelial cells (CEC) and connective tissue (CNT), as shown by a brown cell (Bc). (**B**–**D**) A lipid vacuole (arrows) and mucous-secreting cells (MSCs) are also shown in the epithelium. (**E**) TUNEL-positive cells (circle) were observed in the mantle epithelium layer, indicated with a black arrow. (**F**) Quantitative analysis of the mantle lesions was performed using the Health Assessment Index (HAI). (**G**) The proportion of TUNEL-positive cells in the mantle tissue. (**H**) The density of MSCs. Statistically significant differences between breeding and farming sites are denoted by asterisks (** *p* < 0.01). Abbreviations: Hp = hyperplasia.

The mantle tissue of *C. belcheri* was composed of a layer of columnar epithelial cells covering a connective tissue matrix (Figure 6A). Histological examination identified MSCs and large vacuoles distributed within the columnar epithelial cell layer of the mantle (Figure 6B–E). Brown cells were found only in the samples from the breeding site (Figure 6C–E). HAI values indicated mild histological damage in oysters from the breeding site, with a mean score of 8.0 ± 4.7, compared with 9.0 ± 4.10 in specimens from the farming site (Figure 6F). TUNEL-positive cells were detected within the mantle epithelium (Figure 6E). Quantitative analysis of TUNEL-positive cells revealed higher percentage of TUNEL-positive cells in oysters from the farming site (1.34 ± 0.14%) than in those from the breeding site (0.69 ± 0.12%) (Figure 6G). However, the density of mucous-secreting cells did not differ significantly between the two sites (Figure 6H).

### 2.5. Oxidative Stress and Antioxidant Capacity Assays

**Figure 7:**
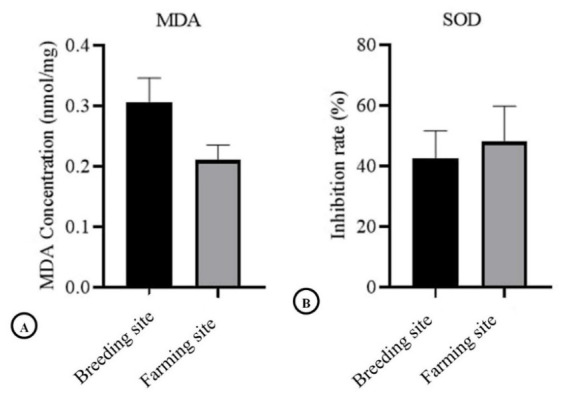
Oxidative stress and antioxidant capacity of oysters from the breeding site and farming site were assessed from malondialdehyde (MDA) levels (**A**) and superoxide dismutase (SOD) activity (**B**) in oyster tissues. The data are presented as means ± SEM.

Excessive ROS generation can induce lipid peroxidation and compromise antioxidant defense mechanisms, leading to increased structural alteration and apoptosis. To evaluate these effects in oyster tissue, MDA concentrations and SOD activities were determined in whole tissues of oysters from both sites. At the breeding site, MDA levels were higher (0.30 ± 0.0 nmol/mg compared with 0.21 ± 0.0 nmol/mg, F = 2.72, *p* > 0.05), and SOD levels were lower (42.55 ± 9.0 compared with 48.10 ± 11.6, F = 1.67, *p* > 0.05). However, the differences were not statistically significant (Figure 7A,B).

## 3. Discussion

*C. belcheri* is an important oyster species for aquaculture in Thailand, recognized as a valuable source of dietary protein for the growing human population. Understanding *C. belcheri* health is a key step toward the sustainable management of its aquaculture, yet detailed investigations remain lacking. In the present study, we conducted the first comprehensive assessment of *C. belcheri* health at the breeding and farming sites.

The gills of bivalves are the vital organs that are in direct contact with water and the surrounding environment [6,7]. The complex arrangement of the gill lamellae in bivalves makes them highly susceptible to damage from environmental stressors and toxic substances [8,10]; it therefore serves as a sensitive marker of health status [6,7]. Our histopathological investigation revealed that the gill of *C. belcheri* showed lamellar fusion in samples from both breeding and farming sites. The HAI of the samples from the farming site was slightly higher than that from the breeding site. Samples from the farming site also showed a higher percentage of TUNEL-positive cells, but there was no clear threshold for TUNEL-positive cell numbers that indicated a health crisis in oysters.

We used the *dad1* gene as a biomarker of stress because it encodes the Defender Against Apoptotic Cell Death 1 protein, a highly conserved subunit of the oligosaccharyltransferase complex involved in N-linked glycosylation in the endoplasmic reticulum (ER) [32,33]. This glycosylation process is essential for proper protein folding and ER homeostasis, making DAD1 a critical regulator of cell survival. Loss or downregulation of *dad1* disrupts ER function, leading to the accumulation of misfolded proteins, activation of the unfolded protein response, and ultimately apoptosis [34]. Given its role in maintaining cellular integrity, *dad1* is often regarded as a stress-responsive gene. Environmental stressors, including temperature fluctuations, salinity changes, pollutants, and pathogenic exposure, have also been shown to modulate *dad1* expression in aquatic species [34]. As found in the present study, there was no significant difference in the relative expression of *dad1* in gill tissue between *C. belcheri* from the two sites, suggesting that environmental parameters at the different sites did not induce substantial physiological stress or activate the ER stress pathway in the oyster. Because only *dad1* expression was examined in this study, further analysis of additional stress- and apoptosis-related genes would be required to fully evaluate physiological stress or ER stress pathway activation in *C. belcheri*.

The level of malondialdehyde (MDA), a stable product of lipid peroxidation, is widely used as a chronic biomarker of oxidative damage in marine invertebrates, such as *Patinopecten yessoensis* [35]. Superoxide dismutase (SOD), a group of metalloenzymes, is an antioxidant enzyme that reflects the physiological defense capacity [36,37,38]. In the present study, MDA levels did not significantly differ between the two sites, consistent with the histopathological findings. Although the difference was not statistically significant, the MDA level at the breeding site was higher than at the farming site, consistent with higher HAI and TUNEL-positive cell levels. These results suggest that oyster populations from the breeding site might be exposed to environmental stressors [39,40,41], although there was no significant difference in *dad1* expression. The underlying mechanism of this question has remained unclear and requires further investigation to clarify the relationship between *dad1* expression and apoptosis levels in *C. belcheri*.

There is considerable evidence that brown cells serve as biological markers of biotic and abiotic stress in bivalves [42,43,44,45]. These cells contain several brown granules in membrane-bound lysosomes, which play a crucial role in detoxifying waste products and degrading foreign material [46,47]. Our results indicated that brown cells in the digestive gland were more abundant in *C. belcheri* from the breeding site, suggesting that the sampled oysters were under stress. These features were also found by Battistini et al. [43], where an increasing number of brown cells were observed in bivalves suffering a severe stress environment and a parasite infection, suggesting their potential role in the immune response of the organism, similar to findings in other bivalves [11,48,49]. Notably, the presence of brown cells in this study may be associated with the unsuitable environmental conditions within the breeding site.

Lipofuscin, a brown pigment, is reported to be an important oxidant source in senescent cells [50]. It is composed of structurally altered lysosomes in the epithelial cells of digestive diverticula following exposure to xenobiotic substances [51,52]. Lipofuscin increases vulnerability to oxidative stress by impairing lysosomal activity [53] and inhibiting the 20S proteasome. Baldensperger et al. [54] noted that lipofuscin accumulation was associated with increased ROS and lysosomal dysfunction. Although the occurrence of lipofuscin in oysters is unclear, we suggest that the high MDA in *C. belcheri* from the breeding site might be related to the presence of lipofuscin accumulation. It can therefore be assumed that the digestive gland of breeding oyster populations is affected, with implications for reduced health, including digestion and/or enzymatic activity. As this study has shown, the mechanisms underlying lipofuscin accumulation must be elucidated to understand its toxicity and its role in cell functional impairment.

## 4. Materials and Methods

### 4.1. Hatchery-Based Oyster Cultivation

Sexually mature *C. belcheri* individuals (about 8 cm shell length, n = 20 individuals) were cultured in the suspended culture box (stocking density = 378.8 individuals/m^2^) at the Marine Shellfish Breeding Research Unit, Faculty of Science and Fisheries Technology, Rajamangala University of Technology Srivijaya (RUTS), Trang, Thailand. The minimal samples were correctly calculated by RStudio (V. 4.3.3) (Alpha = 0.05, Delta = 41.68, Sigma = 64.74 and Power > 80). All specimens were fed an algal mixture composed of *Chaetoceros calcitrans* and *Tetraselmis suecica* at a feeding rate of 6% (dry algal weight per dry oyster meat weight). They were reared in a semi-closed recirculating aquaculture system (RAS) using 0.96 m^3^ fiberglass tanks filled with sand-filtered seawater. Water was recirculated using a submersible pump that generated an upward flow, ensuring continuous flushing of suspended particles. Water passed through a cloth filter at the base of the inner tank, while excess water was discharged through an overflow outlet. Flow rate was maintained at approximately 4 L/min, following the general guidelines of Tanyaros et al. [25], with daily monitoring of water temperature using a multiparameter probe (LAQUA 200 Series Handheld Water Quality Meters, HORIBA Instruments, Singapore) and salinity using a salinometer (Atago MASTER-URC/NM Clinical Refractometer, LEGA, Tokyo, Japan). The pH, nitrite, ammonia, DO and total alkalinity were also recorded. All samples were used for further observations.

### 4.2. Field Sampling and Study Sites

Farmed *C. belcheri* specimens (6–8 cm shell length, n = 20 individuals, Alpha = 0.05, Delta = 41.68, Sigma = 64.74 and Power > 80) were obtained from the suspended culture box (the total area was 1584 cm^2^ and stock density = 60 samples), a coastal aquaculture site in Trang Province, Thailand. The minimal samples were correctly calculated by RStudio (Alpha = 0.05 and Power > 80). These sampled oysters were received from the breeding site as mentioned above since they were at the juvenile stage (about 3–4 cm shell length). All live oysters were used for further processing and analysis. Water temperature, salinity, pH, nitrite, ammonia, DO and total alkalinity were also recorded using the instruments described above.

### 4.3. Morphology and Histological Examination

Oysters from both sites were euthanized according to the AVMA Guidelines for aquatic invertebrates [27]. Fresh tissue samples were resected and dissected into three sub-samples. Some gill tissues (0.5 × 0.5 cm) were fixed from each sample in 2.5% glutaraldehyde for 24 h at 4 °C to investigate the ultrastructure. Other tissue samples were fixed in 10% neutral buffered formalin for 24 h at room temperature and thereafter stored in 70% ethanol. All fixed samples were decalcified with a standard decalcifying solution before paraffin-embedding. Tissue sections at 4 µm thickness were prepared using a rotary microtome and stained with hematoxylin and eosin (H&E) to investigate their structure and histopathology. Histological images of the gill, digestive gland, and mantle were taken using a Panoramic Viewer system (3DHISTECH, Budapest, Hungary) under a light microscope. To assess the health status of individual specimens, the quantitative data for the Health Assessment Index (HAI) for each oyster was examined according to Adams et al. [28]. The HAI value was calculated for each specimen using the formula HAI = (1 × SI) + (10 × SII) + (100 × SIII), where SI, SII, and SIII represent the number of alterations observed at stages I, II, and III, respectively. This weighted scoring system reflects the severity of histopathological changes. HAI values were interpreted as follows: 0–10: normal organ function; 11–20: slight damage; 21–50: moderate damage; 51–100: severe lesions; >100: irreparable lesions [28].

### 4.4. TUNEL-Positive Cell Identification

The TUNEL assay described by Rojo and Gonzalez [29] was used to detect TUNEL-positive cells. Positive tissues were based on those previously reported in our published data [3,55]. Serial tissue sections obtained as described above were dehydrated and incubated in a solution containing 10% normal goat serum (Vector Laboratories, Newark, CA, USA) and 4% bovine serum albumin. The sections were then incubated for 1 h in TdT buffer at 37 °C. After routine processing, TUNEL-positive cells were identified and quantified using ImageJ software (V. 1.54i) by randomly selecting 210 cells per section at 40× magnification, following Ceylan and Kaptaner [30].

### 4.5. Gill Ultrastructure Observations

Gill tissues of *C. belcheri* (n = 3 samples per site), fixed as described in Section 4.3, were post-fixed in 1% osmium tetroxide for 1 h. They were then dehydrated through a graded series of solvents and embedded in Araldite^®^ (Sigma Company, St. Louis, MI, USA). Three ultrathin sections (~90 nm in thickness) per sample were prepared and subsequently examined under a TEM (Philips/FEI Tecnai G2 F20, FEI Co., Eindhoven, The Netherlands).

### 4.6. Molecular Analysis and RT-qPCR of dad1 from Gill Tissues

Gill tissues were excised and trimmed into approximately equal-sized fragments 1–5 mm in length. Total RNA was extracted using GENEzol™ Reagent (Geneaid Biotech Ltd., New Taipei City, Taiwan) according to the manufacturer’s protocol, following homogenization with a tissue homogenizer. RNA concentration and purity were determined using the BioSpectrometer^®^ D30 (Eppendorf, Hamburg, Germany). Total RNA was reverse-transcribed into complementary DNA (cDNA) using RevertAid Reverse Transcriptase (Thermo Scientific, Waltham, MA, USA) according to the manufacturer’s protocol. Each 20 µL reaction contained 1 µg of RNA, 1× RevertAid Reaction Buffer, 0.5 mM dNTPs, 5 µM random hexamers, and 200 U of RevertAid Reverse Transcriptase. The thermal profile included incubation at 25 °C for 10 min, 42 °C for 60 min, 70 °C for 10 min, and a final hold at 4 °C.

The synthesized cDNA was subsequently used as the template for PCR amplification of the gene of interest. The primer sequences used for PCR are listed in Table 2. Each 12.5 µL PCR reaction consisted of 0.5 µL cDNA template, 0.2 mM dNTPs, 1× HF buffer, 0.25 µM each of forward and reverse primers for *dad1* or *β-actin*, and 0.25 U of Phusion™ High-Fidelity DNA Polymerase (Thermo Scientific, Waltham, MA, USA). Thermal cycling was conducted as follows: initial denaturation at 98 °C for 2 min, followed by 35 cycles of denaturation at 98 °C for 10 s, annealing at 56 °C for 30 s, and extension at 72 °C for 35 s, with a final extension at 72 °C for 10 min.

PCR products were purified using the Monarch^®^ DNA Gel Extraction Kit (New England Biolabs, Ipswich, MA, USA) and ligated into the pJET1.2/blunt vector using the CloneJET PCR Cloning Kit (Thermo Scientific, Waltham, MA, USA). Recombinant plasmids were transformed into *E. coli* DH5α cells via heat shock and plated on LB agar containing 100 µg/mL ampicillin (T.P. Drug Laboratories, Bangkok, Thailand). Positive transformants were identified by colony PCR using gene-specific primers. Three confirmed clones were cultured for 16 h with agitation at 200 rpm in LB broth supplemented with ampicillin at 37 °C. Plasmids were extracted using the TIANprep Rapid Mini Plasmid Kit (Tiangen, Beijing, China) and submitted for sequencing (Macrogen, Seoul, Republic of Korea). The resulting nucleotide sequences were analyzed using BioEdit (version 7.2.5) and compared against public databases using BLAST (https://blast.ncbi.nlm.nih.gov) to confirm gene identity.

Expression profiles of the *dad1* gene in *C. belcheri* specimens from both sites (n = 6 per site) were assessed by using reverse transcription quantitative PCR (RT-qPCR). Total RNA was isolated from gill tissue, and cDNA was synthesized as described above. RT-qPCR was carried out using the CFX96 Touch Real-Time PCR Detection System (Bio-Rad, Hercules, CA, USA). Each 20 µL reaction contained 1 µL of cDNA, 1× iQ™ SYBR^®^ Green Supermix (Bio-Rad, Hercules, CA, USA), and 0.2 µM each of forward (qdad1-F) and reverse (qdad1-R) primers. The thermal cycling conditions were as follows: initial denaturation at 95 °C for 3 min, followed by 40 cycles of 95 °C for 15 s, 56 °C for 30 s, and 72 °C for 30 s. A melting curve analysis was included at the end of the amplification protocol to confirm specificity and the absence of non-specific products. Relative gene expression levels were calculated using the 2^−ΔΔCt^ method [31], with *β-actin* serving as the reference gene.

The nucleotide sequence of the cloned partial *dad1* fragment obtained from *C. belcheri* was compared with homologous *dad1* sequences from representative molluscan species available in the NCBI GenBank database. Homologous sequences were identified using BLASTn program available through the National Center for Biotechnology Information (NCBI) BLAST web interface (https://blast.ncbi.nlm.nih.gov/Blast.cgi, accessed on 7 June 2026) against the NCBI nucleotide database (https://www.ncbi.nlm.nih.gov/nucleotide/, accessed on 7 June 2026). Sequences were selected based on sequence similarity and taxonomic representation. Multiple sequence alignment was performed in MEGA X (version 12.1.2). The aligned sequences were manually inspected, and all sequences were trimmed to the region corresponding to the *C. belcheri dad1* fragment prior to phylogenetic analysis. A phylogenetic tree was constructed in MEGA X using the Neighbor-Joining method based on evolutionary distances calculated with the Maximum Composite Likelihood model. The reliability of each node was evaluated using 1000 bootstrap replicates. A homologous *dad1* sequence from *Bemisia tabaci* was included as a non-molluscan outgroup. The resulting tree was used to assess the phylogenetic placement of the cloned *C. belcheri dad1* fragment among molluscan *dad1* homologs.

### 4.7. Oxidative Stress and Antioxidant Capacity Assays

Lipid peroxidation levels in oyster tissue homogenates were determined by quantifying MDA concentrations using a commercial MDA assay kit (Sigma-Aldrich, Darmstadt, Germany), following the manufacturer’s instructions. Briefly, oyster tissues from each experimental group were homogenized in microtubes, incubated with thiobarbituric acid (TBA) at 95 °C for 60 min, and subsequently cooled to −20 °C for 10 min. The formation of a pink chromogen, representing MDA–TBA adducts, was quantified spectrophotometrically at 532 nm. MDA concentrations were expressed as nmol/mg protein. The total protein content of tissue samples was determined using the Bradford method. SOD activity was evaluated using a commercial SOD assay kit (Sigma-Aldrich, Darmstadt, Germany). Oyster tissues were homogenized in 50 mM Tris-HCl buffer (pH 7.4) and centrifuged at 13,000× *g* for 10 min at 4 °C. The resulting supernatant was collected for enzymatic activity determination. The absorbance of the yellow formazan product was measured at 450 nm, and SOD activity was expressed as units per mg protein (U/mg protein).

### 4.8. Statistical Analysis

The water quality parameters, morphometric measurements, HAI values, and the density of TUNEL-positive cells were expressed as means ± standard error (SE). Statistical analysis was performed using GraphPad Prism version 10.4.1 (GraphPad Software Inc., San Diego, CA, USA). Differences between the two experimental groups were assessed using unpaired *t*-tests. Statistical threshold: *p* < 0.05, *p* < 0.001, *p* < 0.0001.

## 5. Conclusions

This study provides the first integrated assessment of health-related histological and cellular responses in *C. belcheri* from breeding and farming sites. The findings indicate that oysters from the two sites exhibited different stress-associated profiles. Oysters from the farming site showed higher proportions of TUNEL-positive cells in the gill and mantle, whereas oysters from the breeding site showed brown cell accumulation and evidence of mild tissue alteration. These results suggest that site-specific environmental and husbandry conditions may differentially influence oyster health. Improvements in stocking density, water-flow management, and waste control should be considered to support sustainable *C. belcheri* aquaculture.

## Figures and Tables

**Table 1:** Water quality parameters of *Crassostrea belcheri* between breeding and farming sites.

Sites (Mean ± SE)	Water Temperature (°C)	Salinity (ppt)	pH	Nitrite (mg/L)	Ammonia (mg/L)	DO (mg/L)	Total Alkalinity (mg/L)
Breeding	28.32 ± 0.37	25.00 ± 0.00	7.69 ± 0.08	0.10 ± 0.00	0.25 ± 0.00	7.69 ± 0.08	136.00 ± 2.83
Farming	31.20 ± 0.10 ****	27.89 ± 0.50 ****	7.43 ± 0.02 *	0.10 ± 0.00	0.23 ± 0.02	7.43 ± 0.02 *	130.30 ± 3.75

Statistically significant differences between breeding and farming sites are denoted by asterisks (* *p* < 0.05, **** *p* < 0.0001).

**Table 2:** Primer sequences used for amplification, cloning, and RT-qPCR analysis of *dad1* and *β-actin* genes in *Crassostrea belcheri*.

Gene	Purpose	Primer Name	Sequence (5′–3′)
*dad1*	PCR amplification/cloning	dad1-F	WTTYCTBTCTTGGTTTCATCTC
*dad1*	PCR amplification/cloning	dad1-R	TCWGCAAADGCTCTTTCTGG
*β-actin*	PCR amplification/control gene	actin-F	GTRCTGTCCCTGTACGCTTC
*β-actin*	PCR amplification/control gene	actin-R	ATGGTGATGACCTGACCGTC
*dad1*	RT-qPCR target gene	qdad1-F	TCTCAACTGTCGGTTCCTTTG
*dad1*	RT-qPCR target gene	qdad1-R	TGCTCTTTCTGGGCTGATTC
*β-actin*	RT-qPCR reference gene	qactin-F	GTRCTGTCCCTGTACGCTTC
*β-actin*	RT-qPCR reference gene	qactin-R	GAAGGGCGTAACCCTCGTAG

## Data Availability

The original contributions presented in this study are included in the article. Further inquiries can be directed to the corresponding author.

## Abbreviations

The following abbreviations are used in this manuscript:

TUNEL	Terminal deoxynucleotidyl transferase dUTP Nick-End Labeling
HAI	Health Assessment Index
ROS	reactive oxygen species
MDA	Malondialdehyde
SOD	superoxide dismutase
*dad1*	defender against apoptotic death 1 gene
TEM	transmission electron microscope
H&E	hematoxylin and eosin
TBA	thiobarbituric acid
